# Beneficial Effects of Nitric Oxide Induced Mild Oxidative Stress on Post-Thawed Bull Semen Quality

**DOI:** 10.22074/ijfs.2015.4244

**Published:** 2015-07-27

**Authors:** Mohsen Sharafi, Mahdi Zhandi, Abdolhossein Shahverdi, Malak Shakeri

**Affiliations:** 1 Department of Animal Science, College of Agriculture and Natural Resources, University of Tehran, Karaj, Iran; 2 Department of Embryology at Reproduction Biomedicine Research Center, Royan Institute for Reproductive Biomedicine, ACER, Tehran, Iran

**Keywords:** Bull, Cryopreservation, Nitric Oxide, Oxidative Stress, Sperm

## Abstract

**Background:**

Cryopreservation of semen requires optimized conditions to minimize the harmful effects of various stresses. The main approach for protection of sperm against stress is based on the use of antioxidants and cryoprotectants, which are described as defensive methods. Recently, the application of controlled mild stressors has been de- scribed for activation of a temporary response in oocyte, embryo and somatic cells. In this study a sub-lethal oxidative stress induced by precise concentrations of nitric oxide (NO) has been evaluated for sperm during cryopreservation.

**Materials and Methods:**

In this experimental study, we used different concentrations of NO [0 µM (NO-0), 0.01 µM (NO-0.01), 0.1 µM (NO-0.1), 1 µM (NO-1), 10 µM (NO-10) and 100 µM (NO-100)] during cryopreservation of bull semen. Their effects on post-thawed sperm quality that included motility and velocity parameters, plasma mem- brane functionality, acrosome integrity, apoptosis status, mitochondrial activity and lipid peroxidation after freezing-thawing were investigated.

**Results:**

Exposure of sperm before freezing to NO-1 significantly increased total motility (88.4 ± 2.8%), progressive motility (50.4 ± 3.2%) and average path velocity (VAP, 53.8 ± 3.1 µm/s) compared to other extenders. In addition, NO-1 significantly increased plasma mem- brane functionality (89.3 ± 2.9%) compared to NO-0 (75.3 ± 2.9%), NO-0.01 (78.3 ± 2.9%), NO-0.1 (76.4 ± 2.9%), NO-10 (64 ± 2.9%) and NO-100 (42 ± 2.9%). Sperm exposed to NO-1 produced the highest percentage of viable (85.6 ± 2.3%) and the lowest percentage of apoptotic (10.8 ± 2.4%) spermatozoa compared to the other extenders. Also, NO-100 resulted in a higher percentage of dead spermatozoa (27.1 ± 2.7%) compared to the other extenders. In terms of mitochondrial activity, there was no significant difference among NO-0 (53.4 ± 3.2), NO-0.01 (52.1 ± 3.2), NO-0.1 (50.8 ± 3.2) and NO-1 (53.1 ± 3.2). For acrosome integrity, no significant different was observed in sperm exposed to different concentrations of NO.

**Conclusion:**

Induction of sub-lethal oxidative stress with 1 µM NO would be beneficial for cryopreservation of bull semen.

## Introduction

The modern cattle industry is interested in improving the ability of cryopreserved semen for oocyte fertilization (1). However, during laboratory manipulation of sperm for the cryopreservation process, various factors such as oxidative, temperature and osmotic stresses lead to reduced sperm fertility (2, 3). This reduction results from damage to the sperms’ integrity due to anatomical and biochemical destruction of subcellular organelles (4). Therefore, it is necessary to use a strategy that opposes these destructive events (5). The common approach used during the last 20 years has been a defensive one based on the use of reagents that contain antioxidant properties such as catalase, superoxide dismutase and glutathione peroxidase as well as cryoprotectants such as egg yolk or soybean lecithin that protect sperm (4, 6, 7).

In recent years, a reported novel approach has led to improvements in the resistance of oocyte, embryo and sperm cells (8-11). The hypothesis for this approach is the use of a mild sub-lethal stress that will enable cells to improve tolerance to a future stress event such as cryopreservation (12). High Hydrostatic pressure (HHP), osmotic stress and oxidative challenges are the main stressors which have been applied for this purpose (13- 15). Sub-lethal HHP has been applied to semen, oocyte, embryo and embryonic stem cells. There was a beneficial effect observed after controlled exposure of these materials to HHP (13). HHP treatment of sperm has been shown to increase the production of special proteins such as the ubiquinol- cytochrome C reductase complex which are thought to play an important role in the fertilization process (16).

Recently, oxidative sub-lethal stress has been reported to induce a temporary resistance to different future stresses. This response is mediated by several physiological pathways, which lead to regulation of programmed cell death (apoptosis) or necrosis (17). Vandaele et al. (15) have reported positive effects of short-term exposure of cumulus oocyte complexes to 50- 100 μM H_2_O_2_. The rate of embryo development parameters significantly improved after *in vitro* fertilization compared to the absence of H_2_O_2_ concentrations.

Sperm processing for cryopreservation also introduced an additional source of oxidative stress by producing free radicals which attacked the sperm membrane and increased its susceptibility to lipid peroxidation (18). To date, no investigation has been conducted to determine the effects of oxidative stress on sperm before cryopreservation. Therefore, the purpose of this study was an attempt to find the mild range of this oxidative stress induced by nitric oxide (NO) on the ability of a sperm or semen sample to withstand cryopreservation.

## Materials and Methods

### Chemicals

All chemicals used in this study were purchased from Sigma (St. Louis, MO, USA) and Merck (Darmstadt, Germany). NO was supplied by Sigma Chemical Company (GS-NO, N4148). Approval for the study was given by the Research Ethics Committees of Tehran University and Royan Institute.

### Farm management and semen collection

This experimental study was conducted at the Department of Animal Science, University of Tehran. Semen samples were collected from 6 mature Holstein bulls (Zar Gene AI Stud) using an artificial vagina (43˚C) twice weekly for one month. After collection, ejaculates were transferred to a water bath (37˚C) and subsequently evaluated for color, volume, motility, concentration and morphology. Samples used in the study met the following standards: semen concentration of ≥1.0×10^9^ spermatozoa/mL, motility ≥60% and ≤15% abnormal morphology. Ejaculates were pooled to eliminate individual differences. Finally, the pooled semen was split into six equal aliquots for processing according to treatments.

### Sperm processing and stress treatment before cryopreservation

Aliquots of ejaculate were diluted at room temperature with Optidyl^®^ (Biovet, France) extender that contained different concentrations of NO [0 μM (NO-0), 0.01 μM (NO-0.01), 0.1 μM (NO-0.1), 1 μM (NO-1), 10 μM (NO-10) and 100 μM (NO-100)]. The semen concentration was set at a final concentration of 100×10^6^ spermatozoa/mL. The diluted semen samples in each treatment were gradually cooled and equilibrated at 4˚C for 150 minutes. Semen samples were subsequently aspirated into 0.25 mL French straws (IMV, L’Aigle, France) and sealed with polyvinyl alcohol powder, then cryopreserved in a computerized freezing machine (Digit Cools, IMVs Technologies, L’Aigle Cedex, France) using a previously tested freezing curve (0.1˚C/minute from 4 to-10˚C, 20˚C/ minute from-10˚C to-110˚C, 40˚C/minute from-110˚C to-140˚C) for bull semen.

### Post-thawing evaluation of sperm parameters

#### Computerized analysis of sperm motility

We used Semen Class Analysis software (SCA) to evaluate total motility (%), progressive motility (%), average path velocity (VAP, μm/sec), straight linear velocity (VSL, μm/sec), curvilinear velocity (VCL, μm/sec), amplitude of lateral head displacement (ALH, μm), straightness (STR, %), and linearity (LIN, %). For analysis, a 5 μl sample of diluted semen was added onto a pre-warmed chamber slide (20 μm, Leja 4, Leja Products Luzernestraat B.V., Holland).

### Plasma membrane functionality

Plasma membrane functionality was determined according to the hypo-osmotic swelling (HOS) test (19). The HOS test relies on membrane resistance to loss of permeability barriers under a stress condition of stretching in a hyper-osmotic medium. We performed HOS by mixing 50 μl of semen with 50 μl of a 100 mOsm/kg hypo-osmotic solution [fructose (9 g/L distilled water), sodium citrate (4.9 g/L distilled water)]. This mixture was incubated at 37˚C for 20 minutes. Then, 200 spermatozoa were randomly assessed to determine the percentage of swollen and non-swollen tails visualized under a phase-contrast microscope (×400 magniﬁcation, CKX41, Olympus, Tokyo, Japan).

### Phosphatidylserine translocation assay

For apoptosis status, Annexin-V was used to track phosphatidylserine translocation in the sperm plasma membrane. A commercial PS Detection Kit (IQP, Groningen, The Netherlands) was used according to the manufacturer’s instructions. After washing spermatozoa with calcium buffer and adjusting the concentration of sperm to 1×10^6^, we added 10 μl of Annexin V-FITC to the sperm suspension, which was allowed to incubate for 15 minutes at room temperature. Then, 10 μl of propidium iodide (PI) was added to the sperm suspension and the resultant suspension was analyzed with a FACS Calibur Flow cytometer (Becton Dickinson, San Jose, CA, USA). For each sample, 10000 events were collected and sperm subpopulations classified as follows: i. Live spermatozoa (Annexin-/ PI), ii. Apoptotic spermatozoa (Annexin+/PI) and iii. Dead spermatozoa (PI+).

### Acrosome integrity

Pisum sativum agglutinin (PSA) was used to identify the integrity of the acrosomal region in post-thawed spermatozoa (20). We added 5 μl of the sperm suspension to 100 μl ethanol (purity: 96%). After 15 minutes, 10 μl of the sperm suspension was mixed with 30 μl of PSA on a glass slide. Finally, 200 sperm per slide were counted by a fluorescent microscope (BX51, Olympus) equipped with fluorescence illumination and a FITC filter (excitation at 455-500 nm and emission at 560- 570 nm) at ×400 magnification. Sperm heads that fluoresced green were considered to have intact acrosome and those with no fluorescence were recorded as damaged or disrupted acrosome.

### Mitochondrial activity

We determined mitochondrial activity by combining fluorescent dyes, Rhodamine 123 (R123, Invitrogen TM, Eugene, OR, USA) and PI. R123 (5 μL) solution was added to 250 μl of diluted semen and incubated for 30 minutes at room temperature in the dark. Then, 5 μl of the PI solution was added to the sample and analyzed with a FACS Calibur Flow cytometer (Becton Dickinson, San Jose, CA, USA). Sperm were analyzed according to their green and red fluorescence stain with R123 and PI. The percentages of live spermatozoa with active functional mitochondria were identified in the R123+/PI− quadrant. For each sample we collected 10000 events.

### Malondialdehyde production

The amount of malondialdehyde (MDA) in the semen samples, as an index of lipid peroxidation, was measured with the thiobarbituric acid reaction (21). MDA concentration was determined by absorption with a standard curve of MDA equivalent generated by the acid catalyzed hydrolysis of 1, 1, 3, 3-tetramethoxypropane.

### Statistical analysis

All data were analyzed using Proc GLM of SAS 9.1 (SAS Institute, version 9.1, 2002, Cary, NC, USA) to determine the effect of different concentrations of NO on post-thawing quality of bull semen. The results were expressed as mean ± SEM. The mean of the treatments were compared using Tukey’s tests.

## Results

### Computerized analysis of sperm motility

Table 1 shows the mean percentage of motility and velocity parameters of the post-thawed sperm exposed to different NO concentrations. NO-1 significantly improved total motility (88.4 ± 2.8) compared to NO-0 (72.5 ± 2.8), NO-0.01 (71.8 ± 2.8), NO-0.1 (79.3 ± 2.8), NO-10 (54.2 ± 2.8), and NO-100 (37.1 ± 2.8). NO-1 also significantly improved progressive motility (50.4 ± 3.2) compared to NO-0 (40.7 ± 3.2), NO-0.01 (41.3 ± 3.2), NO-0.1 (42 ± 3.2), NO-10 (32.6 ± 3.2) and NO-100 (15.7 ± 3.2). NO-1 resulted in significantly higher VAP (53.8 ± 3.1 μm/s) and VSL (40.5 ± 4.2 μm/s) rates compared to the other NO concentrations. There were no significant differences observed between NO-0, NO-0.01, NO-0.1 and NO-1 for VCL and STR. Also, different NO concentrations had no significant effect on the percentage of LIN.

### Plasma membrane functionality

**Fig.1:**
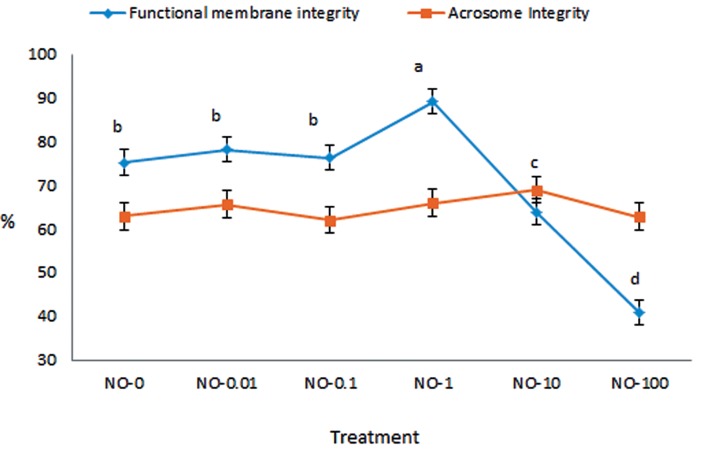
The effect of nitric oxide (NO) induced oxidative stress on plasma membrane functionality and acrosome integrity of post-thawed bull sperm (mean ± SEM). Different letters within the same line show significant differences among the groups (P≤0.05).

Figure 1 shows the alteration in post-thawed sperm plasma membrane functionality and acrosome integrity in different extenders. Plasma membrane functionality showed a similar trend as motility. NO-1 significantly improved plasma membrane functionality (89.3 ± 2.9%) compared to the other extenders. No significant differences were observed among the NO concentrations for the percentage of acrosome integrity.

### Phosphatidylserine translocation assay

**Fig.2:**
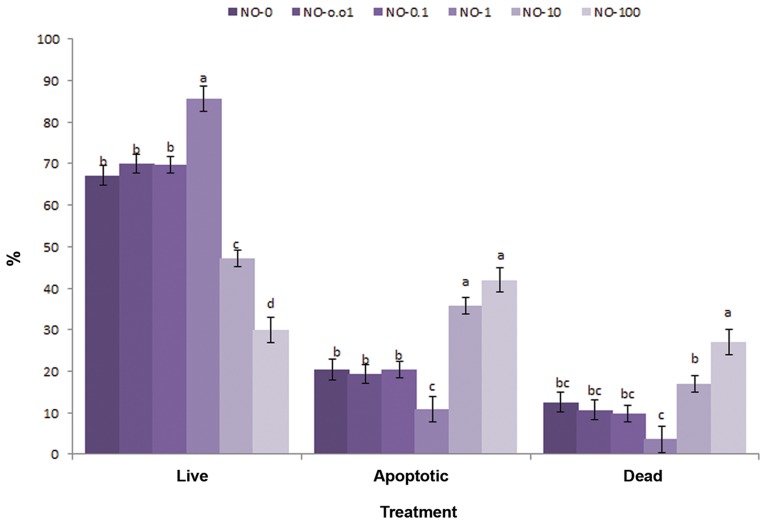
The effect of nitric oxide (NO) induced oxidative stress on the percentage of viable, apoptotic and dead post-thawed bull spermatozoa (mean ± SEM). Viability was assessed by Annexin V and propidium iodide. Different letters within the same column show significant differences among the groups (P≤0.05).

Results of apoptosis status are shown in figure 2. Sperm exposed to NO-1 produced the highest percentage of viable spermatozoa (Annexin-/PI-85.6 ± 2.3%) and the lowest percentage of apoptotic spermatozoa (Annexin+/PI-10.8 ± 2.4%) compared to other NO concentrations. NO-1 (3.6 ± 2.7%) produced a lower percentage of dead spermatozoa compared to NO-10 (17 ± 2.7%) and NO- 100 (27.1 ± 2.7%).

### Mitochondrial activity

**Fig.3:**
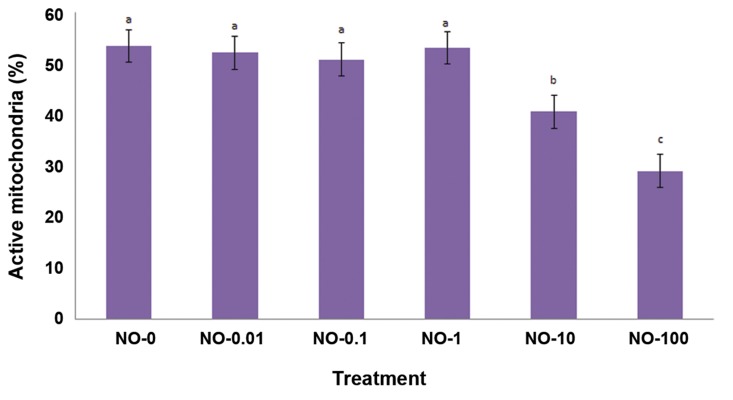
Post-thaw mitochondria potential of bull sperm after oxidative stress by different concentrations of nitric oxide (NO). Different letters within the same column show significant differences among the groups (P≤0.05). Mitochondrial potential was assessed by R123 and propidium iodide.

Figure 3 shows the percentage of post-thawed live spermatozoa with active mitochondria after exposure to different extenders. There were no significant differences between NO-0 (53.4 ± 3.2%), NO-0.01 (52.1 ± 3.2%), NO-0.1 (50.8 ± 3.2%) and NO-1 (53.1 ± 3.2%). However, we observed a significantly lower percentage of live spermatozoa that had active mitochondria in NO-10 (40.6 ± 3.2%) and NO-100 (29 ± 3.2%) compared to the other groups.

### Malondialdehyde production

**Fig.4:**
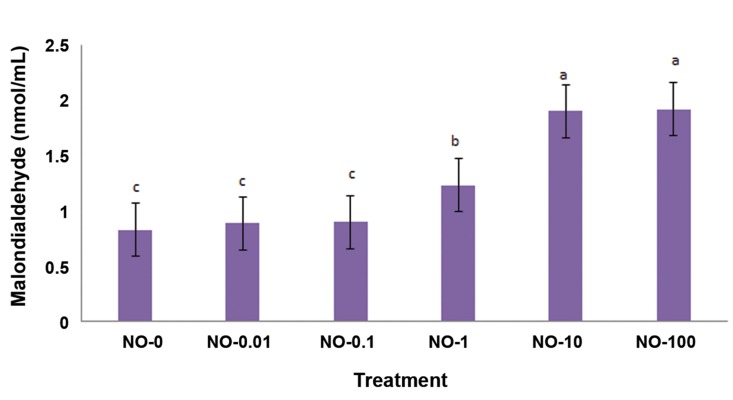
Malondialdehyde (MDA) concentration of frozen-thawed bull sperm after oxidative stress by different concentrations of nitric oxide (NO). Different letters within the same column show significant differences among the groups (P≤0.05). Lipid peroxidation was assessed by MDA assay.

Figure 4 shows the results of MDA concentration as a lipid peroxidation rate. Sperm exposed to NO-1 produced a significantly higher concentration of MDA (1.23 ± 0.24) compared to NO-0 (0.83 ± 0.24), NO-0.01 (0.89 ± 0.24) and NO-0.1 (0.9 ± 0.24). The highest MDA concentrations were observed in NO-10 (1.9 ± 0.24) and NO-100 (1.92 ± 0.24).

**Table 1:** The effect of nitric oxide (NO) induced oxidative stress on motility and velocity parameters of post-thawed bull sperm


Variable	Extenders					
	NO-0	NO-0.01	NO-0.1	NO-1	NO-10	NO-100

Total motility (%)	72.5 ± 2.8 c	71.8 ± 2.8 c	79.3 ± 2.8 b	88.4 ± 2.8 a	54.2 ± 2.8 d	37.1 ± 2.8 e
Progressive motility (%)	40.7 ± 3.2 b	41.3 ± 3.2 b	42 ± 3.2 b	50.4 ± 3.2 a	32.6 ± 3.2 c	15.7 ± 3.2 d
VAP (μm/s)	44 ± 3.1 b	46.1 ± 3.1 b	45.9 ± 3.1 b	53.8 ± 3.1 a	24.2 ± 3.1 c	22.3 ± 3.1 d
VSL (μm/s)	28.1± 4.2 b	29.3 ± 4.2 b	39.1 ± 4.2 a	40.5 ± 4.2 a	26.4 ± 4.2 b	27.1 ± 4.2 b
VCL (μm/s)	60.5 ± 3.8 a	63.75 ± 3.8 a	64.62 ± 3.8 a	65.41 ± 3.8 a	62.8 ± 3.8 a	34.7 ± 4.8 b
STR (%)	70.4 ± 2.9 a	68.7 ± 2.9 a	71.1 ± 2.9 a	72.7 ± 2.9 a	53.8 ± 2.9 b	54 ± 2.9 b
LIN (%)	38.3 ± 1.4	37.6 ± 1.4	37 ± 1.4	39.5 ± 1.4	38.4 ± 1.4	37.2 ± 1.4


Different letters within the same row show significant differences among the groups (P≤0.05). VAP; Average path velocity, VSL; Straight linear velocity, VCL; Curvilinear velocity, STR; Straightness and LIN; Linearity.

## Discussion

The results of the present study have shown beneficial effects of NO induced sub-lethal oxidative stress for bull sperm during cryopreservation. During the process of freezing-thawing, spermatozoa are exposed to numerous stressful conditions which can cause disruption of cellular organelles and function (22). This experiment is the first study to investigate the controlled offensive approach by oxidative stress for creating a temporary response against future environmental challenges.

Our trial used a design that created a wide range of oxidative stress (0.01-100 μM NO) to determine the best concentration that could induce temporary resistance in the sperm against more serious stress during cryopreservation. Our analysis showed that the effective range was limited to 0.1-1 μM NO which improved motility, plasma membrane functionality, viability and mitochondria activity. A high amount of MDA production as indices of lipid peroxidation was observed in sperm treated with NO concentrations greater than 1 μM of NO. The present results also demonstrated that high oxidative stress to sperm before freezing produced a high amount of MDA which was directly responsible for lower motility, viability, mitochondria activity and plasma membrane functionality in the groups exposed to greater than 1 μM NO. MDA, as an index of lipid peroxidation, increased during cryopreservation due to high activity of reactive oxygen species (ROS) (23).

The fact that sub-lethal stress increased temporary resistance to future stresses has been observed in different types of live cells (24). Conserved proteins in the cells are the main keys that participate in this process, by repair and stabilization of DNA, proteins and the cytoskeleton (25, 26). 

Production and phosphorylation of heat shock proteins (HSPs) is another reason for enhancement of resistance after sub-lethal stress (26-28). It has been reported that HSPs directly inhibit the intrinsic and extrinsic pathways of apoptosis in the cell (29). Although we did not measure the amount of HSPs after oxidative stress, however it has been shown to increase HSPs level in both prokaryotes and eukaryotes expose to stress condition. In a recent study, phosphorylation of HSPs 70 and 90 in frozen-thawed sperm significantly increased compared to pre-freezing which assisted sperm to oppose stressful conditions.

However, we observed that exposure to oxidative stress over the limit of tolerance increased apoptosis and necrosis. These findings agreed with Hansen (17) who reported that heat stress greater than tolerance of embryonic stem cells led to higher numbers of apoptotic events. HHP is a one of controlled stress for sperm. It has been shown that HHP stress increased survival and fertility of prolonged storage *in vitro* (9, 30, 31). In a study by Kuo et al. (32), mild stress by HHP did not change the pregnancy rate but increased litter size after insemination with post-thawed semen. Huang et al. (16) stated that stress by HHP particularly increased proteins in sperm which played a key role in fertilization.

Similar to HHP stress, we obtained an improvement in bull semen quality by induction of moderate oxidative stress to the sperm before cryopreservation. Our result agreed with the finding by Vandaele et al. (15) who reported that a low level of oxidative stress by 50-100 μM H_2_O2 for oocytes resulted in a higher blastocyst rate after *in vitro* fertilization. This efficiency might be attributed to increasing levels of antioxidants such as GSH in the oocyte which positively affected development.

However, in other studies, oxidative stress resulted in negative effects on the blastocyst rate by increasing the numbers of apoptosis cells (33-35). This discrepancy might be related to various factors such as manipulation of cells, type and level of stress, and cell type (oocyte, embryo sperm or fibroblast). We have also found that mild stress may reduce the apoptosis rate in sperm exposed to 1 μM NO before freezing which shows a logical relationship with motility. Mitochondria play a primary role in extending the phase of apoptosis as a result of the opening of mitochondrial pores which leads to the subsequent release of pro-apoptotic factors. Synthesis of ATP is under the control of mitochondrial activity and damage to mitochondria lead to non-renewal of ATP, this negatively affects the sperms’ ability to move.

## Conclusion

This study has shown that a mild level of oxidative stress treatment (1 μM NO) prior to cryopreservation offers an approach to improve the quality of frozen-thawed semen performance such as motility, viability, plasma membrane functionality and mitochondrial activity. Understanding the molecular and cellular mechanism of this phenomenon needs more investigation. A filed study using this approach along with artificial insemination, *in vitro* fertilization, and the application of different procedures for manipulation of sperm are also necessary.
